# Learning health systems: A review of key topic areas and bibliometric trends

**DOI:** 10.1002/lrh2.10265

**Published:** 2021-03-18

**Authors:** Chiara Pomare, Zeyad Mahmoud, Alex Vedovi, Louise A. Ellis, Gilbert Knaggs, Carolynn L. Smith, Yvonne Zurynski, Jeffrey Braithwaite

**Affiliations:** ^1^ Australian Institute of Health Innovation Macquarie University Sydney Australia; ^2^ Partnership Center for Health System Sustainability Macquarie University Sydney Australia

**Keywords:** bibliometrics, healthcare, learning health systems, learning healthcare systems

## Abstract

**Introduction:**

The emergent field of learning health systems (LHSs) has been rapidly evolving as the concept continues to be embraced by researchers, managers, and clinicians. This paper reports on a scoping review and bibliometric analysis of the LHS literature to identify key topic areas and examine the influence and spread of recent research.

**Methods:**

We conducted a scoping review of LHS literature published between January 2016 and May 2020. The authors extracted publication data (eg, journal, country, authors, citation count, keywords) and reviewed full‐texts to identify: type of study (empirical, non‐empirical, or review), degree of focus (general or specific), and the reference used when defining LHSs.

**Results:**

A total of 272 publications were included in this review. Almost two thirds (65.1%) of the included articles were non‐empirical and over two‐thirds (68.4%) were from authors in the United States. More than half of the publications focused on specific areas, for example: oncology, cardiovascular care, and genomic medicine. Other key topic areas included: ethics, research, quality improvement, and electronic health records. We identified that definitions of the LHS concept are converging; however, many papers focused on data platforms and analytical processes rather than organisational and behavioural factors to support change and learning activities.

**Conclusions:**

The literature on LHSs remains largely theoretical with definitions of LHSs focusing on technical processes to reuse data collected during the clinical process and embedding analysed data back into the system. A shift in the literature to empirical LHS studies with consideration of organisational and human factors is warranted.

## 1 INTRODUCTION

Contemporary health systems are not fit for purpose. Even in the most developed countries less than two‐thirds of healthcare delivered is in line with evidence‐based guidelines (60%); one third of care is some form of waste (30%) and one tenth (10%) of it is associated with an adverse event.^1^ These numbers have persisted for decades despite substantial efforts and resources dedicated to improving the safety and quality of care across the globe.^2^ To overcome what has been coined the “60‐30‐10 Challenge,”^1^ health systems will need to become more dynamic and responsive to new and emerging evidence. The Learning Healthcare model developed by the Institute of Medicine (IoM; now the National Academy of Medicine) outlines the form and function of such dynamic systems.^3^, ^4^, ^5^

A Learning Health System (LHS), as defined by the IoM, is one where “science, informatics, incentives, and culture are aligned for continuous improvement and innovation, with best practices seamlessly embedded in the care process, patients and families active participants in all elements, and new knowledge captured as an integral by‐product of the care experience”.^3^ Although this is clearly an aspirational definition, a realized LHS has the potential to amalgamate data (eg, laboratory reports, patient histories, and cost data) for real‐time decision‐making, which can support the delivery of safe and high‐quality care. Recent advances in technology, including the ability to collect and integrate massive amounts of routinely collected health data, have enabled health systems to begin to realise the potential of an LHS.^6^, ^7^

Although the concept of an LHS is increasingly being embraced as a way to create smarter health systems,^8^ it is more of a journey than a destination, and the evidence base surrounding LHSs is still in the early stages of development.^9^, ^10^ Previous reviews on this topic show that there is growing interest in LHS concepts and ideas, but relatively few empirical applications have been published to date. In 2016, a systematic review of the LHS literature by Budrionis and Bellika^9^ revealed that of the 32 identified papers, only 13 described an empirically operationalised LHS.^9^ Two years later, in 2018, Platt et al^10^ conducted a scoping review and found that the LHS literature remained mostly theoretical, despite the growing number of publications in the LHS domain. Since these reviews, there has been increasing interest in the application of LHS concepts. LHSs are now being developed and adopted at different levels of learning by individuals, teams or networks, organisations, and “ultra‐large‐scale systems”.^11^ Threats to the sustainability of health systems require adaptive health system responses, as aptly demonstrated by the COVID‐19 pandemic.^12^, ^13^ As the LHS field enters its second decade, and the concept continues to be embraced by researchers, managers, and clinicians, it is timely to examine the influence and spread of the LHS literature. The aims of this paper were therefore to summarise key topic areas and to examine bibliometric trends of the most recent LHS literature.

## 2 METHODS

### 2.1 Review methods and eligibility criteria

We carried out a scoping review of LHS literature published between January 2016 and May 2020. A scoping review synthesises and maps the research on a particular topic to identify key concepts and gaps in the literature.^14^ Searches were carried out in two online databases (PubMed and Scopus) using the term “learning health* system*”. Publications were downloaded into EndNote and duplicates were removed. The review team (CP, ZM, AV, LAE, GK, CLS) screened retrieved publications in full to determine their inclusion. Publications were included if they were (a) in the English‐language, (b) peer‐reviewed publications (journal articles, review articles, journal commentaries, editorials, books, or book chapters), and (c) had a key focus on LHS. Publications containing no substantive discussion of the LHS concept (eg, only superficially used the term in the abstract, conclusion, or among the keywords) were excluded. Five percent of retrieved publications were independently screened by the entire review team to ensure consistent inclusion. Disagreements about the eligibility of publications for inclusion were resolved through review team consultation, with YZ and JB resolving any outstanding disagreements.

### 2.2 Data extraction

Publications were reviewed in full to identify publication data (the journal in which the paper was published, keywords, author names, and the country of residence of the corresponding author), the type of study (empirical, non‐empirical, or review), degree of focus (general or specific), and, if a definition was included. This information was extracted and stored in Microsoft Excel 365. The number of citations for each publication was also recorded, as reported by Web of Science in September 2020, and was used as a measure of impact or influence, rather than as an indicator of quality.

### 2.3 Analysis

Publication data were collected to determine the most active journals, authors, and countries publishing in the LHS field. For degree of focus, a publication was coded as “general” when LHS concepts were discussed without discussing a specific program, health condition, or topic area. Publications coded as having a specific degree of focus were later aggregated to create counts per key topic area (ie, to delineate how many included publications focused on ethics). Key topic areas were also examined through an analysis of keywords. Keywords of publications were identified in EndNote X9, then cleaned and checked for consistency. Derivatives (eg, health system, healthcare systems, health systems) were amalgamated. The keyword data were analysed for frequency counts and co‐occurrence using VOSviewer v.16.14 (https://www.vosviewer.com/). The citation average for the field was calculated by citation count per paper divided by the number of years elapsed since publication.

Where a definition of LHS was used, we extracted the definition and referenced citation. Definitions were analysed using text analysis to determine word use frequency (ie, similar terms were grouped together, such as improvement, improve, improving). LHS definition references were graphically presented using Gephi, version 0.9.2. The nodes in the network were the publications in the review, as well as any output that was cited as a definition. Ties were LHS definition citations (eg, publication X cited publication Y to define an LHS). The most influential publications in the network were assessed using in‐degree calculation^15^ (ie, the number of times a reference was cited by different publications when defining an LHS).

## 3 RESULTS

The search yielded 430 publications. After the removal of duplicates, there were 420 publications, 148 of which did not meet the inclusion criteria. This resulted in 272 publications on LHSs included in our analysis.

### 3.1 Bibliometric properties of included publications

Of the 272 publications, most were non‐empirical (n = 177, 65.1%), one quarter were empirical (n = 68, 25.0%), less than a tenth were reviews of the literature (n = 25, 9.2%), and two were books (0.7%). Overall, there was an average of 3.0 citations a year per publication, although there was a high degree of skewness (Min = 0, Max = 32.3).* The most highly cited publication was: Rumsfeld, Joynt, & Maddox, (2016), Big data analytics to improve cardiovascular care: promise and challenges. *Nature Reviews Cardiology*, 13(6), 350‐359. It was cited 129 times (32.3 citations per year). The five most frequently cited publications are in Table 1.

**TABLE 1:** Top cited publications on LHSs (2016‐2020)

Ranking	Authors	Country	Title	Year	Journal	Impact factor	Average citations per year	Total citation count^a^
1	Rumsfeld et al	United States	Big data analytics to improve cardiovascular care: promise and challenges.	2016	*Nature Reviews Cardiology*	20.26	32.3	129
2	Chambers et al	United States	Convergence of implementation science, precision medicine, and the learning health care system: a new model for biomedical research.	2016	*Journal of the American Medical Association* (*JAMA*)	45.54	26.5	106
3	Horwitz et al	United States	Creating a learning health system through rapid‐cycle, randomized testing.	2019	*New England Journal of Medicine*	74.70	15	15
4	Alsheik et al	United States	Comparison of resource utilization and clinical outcomes following screening with digital breast tomosynthesis versus digital mammography: findings from a learning health system.	2019	*Academic Radiology*	2.50	10	10
5	Shah et al	United States	Building a rapid learning health care system for oncology: why CancerLinQ collects identifiable health information to achieve its vision.	2016	*Journal of Clinical Oncology*	32.96	8	32

^a^ Total number of citations in Web of Science as of September 2020.

The most productive authors in the LHS field from 2016 to 2020 were all from the United States: Charles P. Friedman (n = 8), Amy M. Kilbourne (n = 6), and Eugene C. Nelson (n = 6). Authors who have published on LHS in the last four years were from 25 different countries, with most publications coming from the United States (n = 186, 68.4%), followed by Canada (n = 22, 8.1%), and the United Kingdom (n = 17, 6.3%). The most active journals publishing on LHSs were *Learning Health Systems* (n = 15, 5.5%) and *eGEMS* (Generating Evidence & Methods to improve patient outcomes) (n = 15, 5.5%). For the top 10 most active journals on this topic, see Table 2.

**TABLE 2:** Top 10 most‐active journals contributing to the LHS literature (2016‐2020)

Journal	Impact factor	Number of publications
*Learning Health Systems*	Not stated	15
*eGEMS* ^a^	Not stated	15
*Medical Care*	3.21	8
*JAMA*	45.54	7
*Studies in Health Technology and Informatics*	0.44	6
*BMJ Open*	2.50	5
*BMJ Quality and Safety*	7.23	5
*Yearbook of Medical Informatics*	0.83	5
*Journal of Clinical and Translational Science*	3.99	4
*Journal of Biomedical Informatics*	2.95	4

^a^ In 2019, eGEMS transitioned from a free‐standing journal to a special section in *Healthcare*: *The Journal of Delivery Science and Innovation*.

### 3.2 Defining learning health systems

Most publications (n = 193, 70.4%) provided a definition of an LHS. Ninety‐five different citations were used when providing a definition of an LHS. More than half of the definition references were used more than once (n = 56, 58.9%). Seven definitions did not have citations and the wording could not be identified as belonging to a previous paper; these were classified as definitions developed by the author(s). Common terms used when defining LHSs are listed in Table 3. Frequently used terms closely related to the technical processes of generating knowledge from data analysis and embedding it back into the system (eg, improvement, patient, continuously, knowledge, practices). Terms used to indicate organisational and human factors (eg, organisational culture, skills, training, staff buy‐in, patient participation) were less frequently used.

**TABLE 3:** Most common terms used in LHS definitions (2016‐2020)

Phrases and terms	Occurrences^a^
Improvement	165
Patient	141
Data	136
Continuously	126
Knowledge	122
Practices	108
Delivery	91
Research	88
Evidence	86
Process	79
Generate	76
Clinical	76
New	72
Best	71
Integral	62

^a^ Word count based on verbatim language from definitions in 193 papers.

**FIGURE 1:**
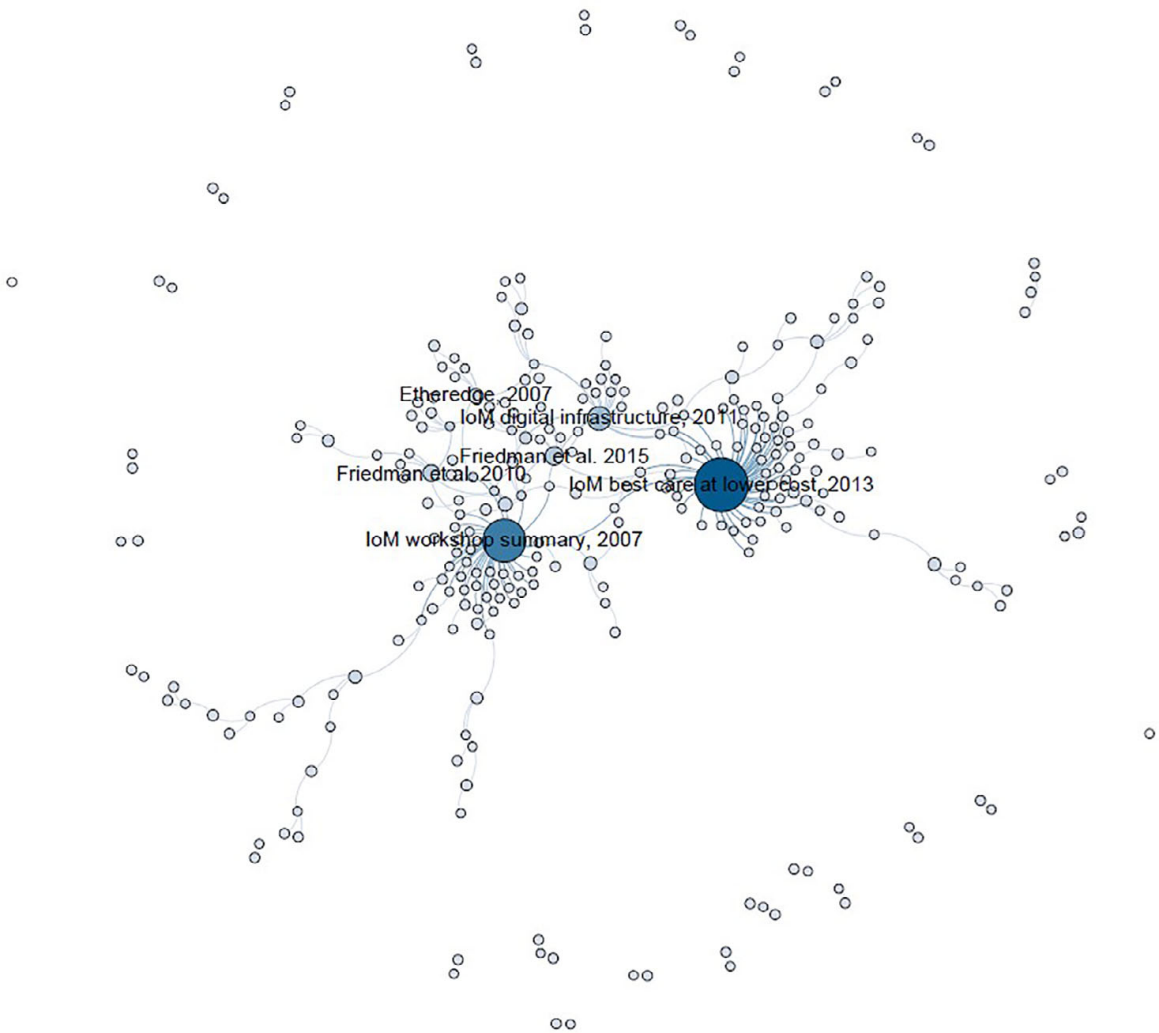
LHS definition network. Each circle (node) is a publication, and each line (tie) represents citation to a publication as the definition reference. Size of node indicates in‐degree (larger nodes indicate a higher number of citations for an LHS definition)

The network of publications and their definition references for LHSs consisted of 273 nodes (publications), and 269 directional ties (citation to a reference) (Figure 1). In this network, there were 28 clusters (ie, groups of connected nodes). The main cluster includes the highly cited definition references (ie, the IoM reports). The IoM's 2013 report *Best care at lower cost*: *The path to continuously learning health care in America* ^3^ was the most frequently cited publication when defining an LHS (cited in connection with 53 LHS definitions). See Table 4 for the most‐cited sources of LHS definitions.

**TABLE 4:** Most‐cited sources of LHS definitions (2016–2020)

Source and references	N^a^
Institute of Medicine^3^	53
Institute of Medicine^4^	46
Institute of Medicine^5^	14
Friedman et al^11^	12
Friedman et al^16^	9
Etheredge^8^	7

^a^ Number of publications in this review that cited the reference as a definition of an LHS.

### 3.3 Key topic areas

**FIGURE 2:**
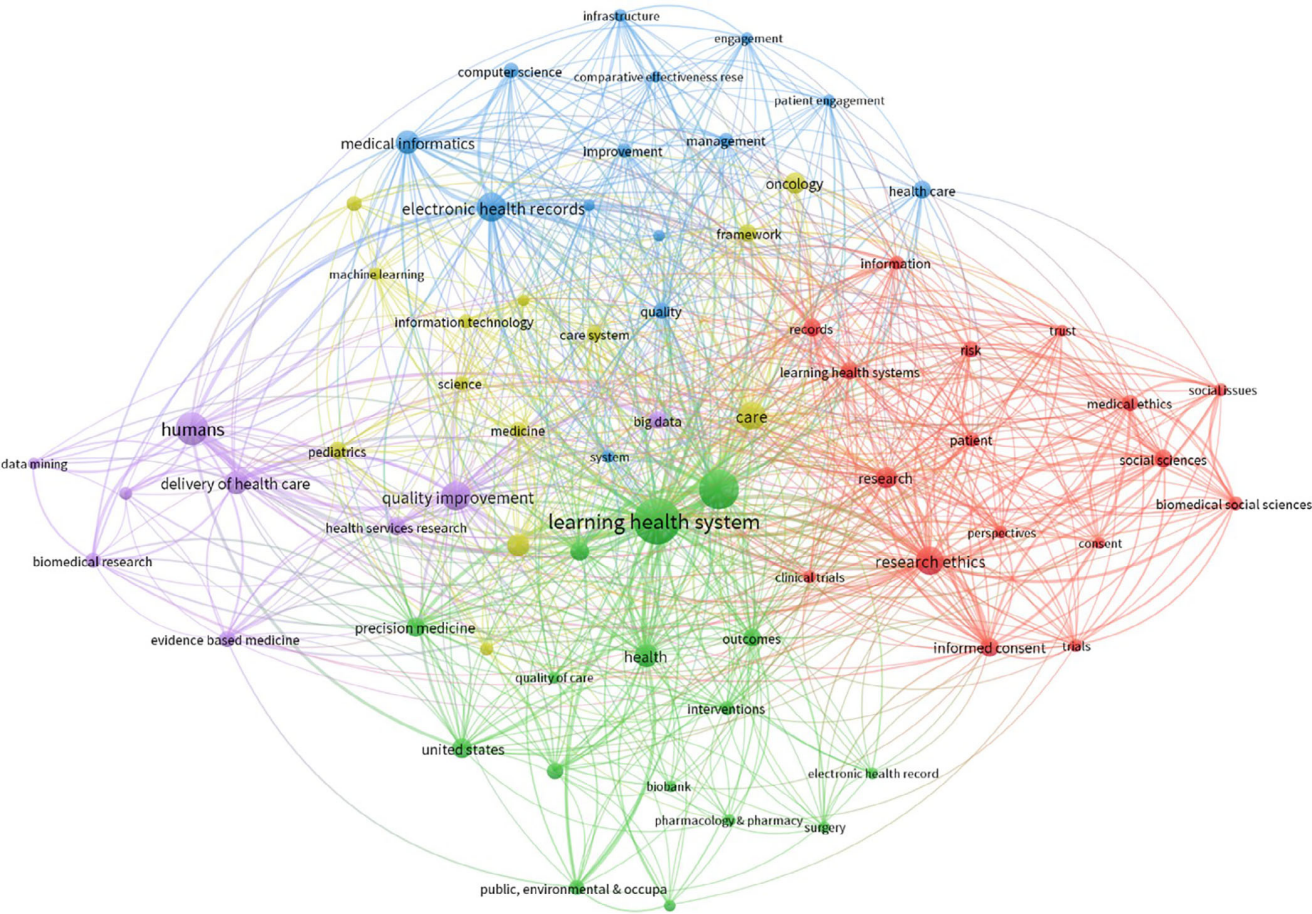
Network of co‐occurring keywords in the recent LHS literature. Each circle (node) is a keyword, and each line (tie) represents co‐occurrence. Size of node indicate the number of times a keyword was used. Colours represent different clusters of keywords

Among the 272 included publications, 1067 unique keywords were identified. Of these, 69 were used at least five times. Co‐occurrence of keywords (ie, keywords used together on a publication) is visually depicted in the network of co‐occurring keywords (Figure 2). In this network, each circle represents a keyword and each line indicates co‐occurrence on a publication. The most frequently used are summarised in Table 5.

**TABLE 5:** List of prominent keywords in the LHS literature (2016‐2020)

Keyword	Occurrences
Learning health system	78
Health care sciences and services	56
Humans	39
Electronic health records	32
Quality improvement	32
Research ethics	28
Medical informatics	21
Delivery of health care	20
General and internal medicine	18
Research	16
Oncology	16

Commonly used keywords were consistent with the key topic areas identified for the included publications. We found that more than half (n = 152, 55.9%) of the included publications had a specific degree of focus (ie, examined a specific program, health condition, or topic through the lens of LHSs), while the remaining 119 (43.8%) explored general aspects of the LHS concept. Specific topics areas included: ethics (n = 16), research and clinical trials (n = 15), oncology (n = 24), cardiovascular care (n = 7), and genomic medicine (n = 7).

## 4 DISCUSSION

In this paper, we summarised key topic areas of the LHS literature and examined bibliometric trends in this rapidly developing area between 2016 and 2020. We identified the most relevant journals, most active authors, and countries that have published in this area, conducted a network analysis of definition references and co‐occurring keywords, and identified key topic areas. In the last 4 years, most (65.1%) of the literature on LHSs has been non‐empirical and the majority (68.4%) has come from the United States.

Similar to a past review on this topic,^10^ we found that the most commonly cited source used to define an LHS was the IoM. One reason is that the IoM has published several different outputs explaining LHS concepts.^3^, ^4^, ^5^ Overall, we found a large degree of convergence around definitions describing what constitutes an LHS. Typically, these definitions referred to an LHS as achieving healthcare quality improvement by using big data and embedding data analysis and intelligent decision‐making into routine care delivery processes. This was consistent with key topic areas identified in our analysis, such as: electronic health records, quality improvement, and medical informatics. This is a promising finding for the emerging LHS field, as developing a common understanding and vocabulary around the LHS concept is imperative for its advancement.^17^ Notwithstanding this, our findings suggest that the definitions and explanations of LHSs focused primarily on the information technology capabilities of an LHS at the expense of human and organisational factors. These human and organisational factors are necessary for environmental, system‐wide change to support LHS development, such as organisational culture and the values and beliefs of staff required to drive behavioural and organisational change.^18^

Another prominent topic area identified in this review was research ethics, such as the ethics of data access and re‐use for clinical efficacy and safety. As the promise and potential of the LHS vision continue to emerge, there is increasing consideration of the ethical challenges that need to be addressed. A notable ethical challenge in LHSs is delineating to what extent structures need to be in place for informed consent^19^ and where the difference lies between clinical care and research.^20^ Publications included in our review referred to an earlier LHS ethics framework conceptualised by Faden et al.^21^ In this framework, practitioners and institutions have an ethical obligation to feed information into the system to increase knowledge, while patients have an ethical obligation to contribute their experiences and health information to research. According to this framework, informed consent should only be sought if the learning activity could pose negative ramifications on care or result in burdens beyond normal expectations. However, access to data is often governed by laws and regulations determined outside of any individual LHS, and in many cases such laws and regulations may result in delayed access or other obstacles to the timely use and reuse of data collected during the course of clinical care provision. Despite a wide‐ranging and robust set of discussions and contributions on the subject, the ethical questions of data use in LHSs are far from settled.

As to progress with implementation, we identified specific healthcare conditions and services that were common sites for the adoption of LHSs. These included: oncology, cardiovascular care, general and internal medicine, and genomic medicine; these are consistent with findings from a past review on LHSs that identified similar clinical contexts.^10^ For example, oncology has become a key setting to examine concepts of and progress with LHSs, with one contributing factor being the implementation of the American Society of Clinical Oncology's CancerLinQ initiative.^22^ CancerLinQ is a large database that collects information from electronic health records of cancer patients to foster an oncology learning community across the United States; it has been described as a rapid LHS.^23^ This example demonstrates the inherent need for integrated data platforms to support the adoption of LHSs and the importance of rapid implementation and implementation science in this sphere.^24^

As to strengths and limitations, this study highlighted key topic areas and summarised the scientific landscape of the last four years of the LHS literature. Our findings can be used to identify gaps in research and guide the next wave of literature on LHS research. Although authors from the United States published most of the literature, this does not necessarily mean that research from other countries is lacking. The LHS concept and terminology were coined by the United State's IoM; therefore, it is not surprising that when searching for LHS in the literature, much of it originates in the United States. There may be equivalent terms used in other parts of the world and in languages other than English. Furthermore, we did not include grey literature which may further broaden our understanding of the LHS field and should be included in future reviews. There is a need to capture research written in languages other than English to obtain a more widespread view of the LHS concept and its potential. This is particularly important given the divide between high‐, middle‐, and low‐income countries when it comes to big data and personalised medicine,^25^ which are key components of LHSs.

## 5 CONCLUSION

Despite ongoing interest in the concept of LHS as demonstrated by the growing body of literature, this study shows that considerations of LHS concepts are largely focused on technical processes rather than the organisational and human factors necessary to facilitate an LHS. The LHS literature warrants a shift in focus to move the field from the conceptual LHS to take‐up and adoption, and from technical processes to emphasising the complexity of human factors in LHSs.

## CONFLICT OF INTEREST

The authors declare that they have no conflicts of interest.

## Endnote

* Publications from 2020 were not included in this calculation.
